# Determinants of survival of the bovine blastocyst to cryopreservation stress: treatment with colony stimulating factor 2 during the morula-to-blastocyst transition and embryo sex

**DOI:** 10.1186/s43170-020-00012-9

**Published:** 2020-09-03

**Authors:** Froylan Sosa, Jeremy Block, Yao Xiao, Peter J. Hansen

**Affiliations:** 1 Department of Animal Sciences, D.H. Barron Reproductive and Perinatal Biology Research Program, and Genetics Institute, University of Florida, Gainesville, FL 32611-0910, USA; 2 Zoetis Inc., Kalamazoo, MI 49007, USA

**Keywords:** CSF2, Blastocyst, Embryo, Vitrification, Bovine, Sex

## Abstract

**Background:**

Colony-stimulating factor 2 (CSF2) is an important maternal regulator of embryonic development. Earlier research indicates that CSF2 can regulate genes involved in cellular stress responses and block apoptosis. Here, we tested whether addition of 10 ng/mL CSF2 at day 5 of development would increase the survival of blastocysts harvested at day 7 and subjected to vitrification. Additional objectives were to determine whether embryo sex affected survival or whether effects of CSF2 interacted with sex.

**Results:**

Survival after vitrification was measured as the percent of warmed blastocysts that re-established a blastocoele after culture and that underwent hatching from the zona pellucida. In the first experiment, blastocysts were vitrified, warmed, cultured for 24 h, and DNA embryo sexing performed by PCR. There was no effect of CSF2, sex, or the interaction on the percent of blastocysts that re-expanded or that were hatching or hatched. In the second experiment, vitrified blastocysts were warmed and cultured for 24, 48, and 72 h. Treatment with CSF2 increased (P = 0.021) the percent of blastocysts that re-expanded as compared to the vehicle group (overall, 77.8 ± 4.7% vs 73.3 ± 4.7%). Percent re-expansion was highest at 24 h and declined slightly thereafter (P = 0.024). Although the interaction was not significant, the effect of CSF2 was greater at 48 and 72 h than at 24 h because CSF2 reduced the incidence of embryos collapsing after re-expansion. Furthermore, the proportion of re-expanded blastocysts at 24 h that experienced blastocoel collapse by 72 h was lower (P = 0.053) for CSF2 (3.6%; 7/195) than for vehicle (8.2%; 16/195). The percent of warmed blastocysts that were hatching or hatched increased with time (P < 0.0001) but there was no effect of CSF2 or the interaction with time on hatching.

**Conclusion:**

Treatment with CSF2 from day 5 to 7 of development did not cause a significant effect on the percent of blastocysts that re-established the blastocoele after 24 h of culture but CSF2 reduced the collapse of the blastocoele that occurred for a portion of the embryos that had experienced re-expansion at 24 h. Thus, CSF2 can provide protection to a proportion of blastocysts from cryodamage caused by vitrification. Further work is needed to evaluate whether CSF2 increases survival of vitrified blastocysts after embryo transfer.

## Background

Among the mechanisms by which the maternal environment regulates development of the mammalian embryo is through the secretion of molecules called embryokines that modify the developmental phenotype of the embryo to affect competence to establish pregnancy and postnatal function (Hansen and Tríbulo 2019). One such embryokine in the cow is colony stimulating factor 2 (CSF2). This cytokine, which is produced by the uterine endometrium (Moraes et al. 1999; Tríbulo et al. 2018), can act on the bovine embryo from day 5 to 7 of development (a time coincident with the morula to blastocyst transition) to increase the competence of the embryo to reach the blastocyst stage in vitro (Moraes and Hansen 1997; Loureiro et al. 2009; Dobbs et al. 2013; Siqueira and Hansen 2016), modify trophoblast elongation (Dobbs et al. 2014), and increase embryo pregnancy rate after transfer to recipients (Dobbs et al. 2013; Denicol et al. 2014). There is one report that calves produced from embryos treated with CSF2 experience greater postnatal growth than calves from control embryos (Kannampuzha-Francis et al. 2015).

It is likely that an important aspect of CSF2 actions on development of the preimplantation embryo is its role as a survival factor to reduce the adverse effects of stress on the embryo. Treatment with CSF2 reduced the induction of apoptosis in Day 6 bovine embryos caused by heat shock (Loureiro et al. 2011). Moreover, a large fraction of the 635 genes whose expression was regulated by CSF2 in female blastocysts are involved in processes related to cell stress (Zolini et al. 2020). In the mouse, too, CSF2 treatment can reduce expression of genes involved in apoptosis and the heat shock protein response (Chin et al. 2009).

Another important determinant of the function of the preimplantation embryo is embryo sex. In cattle, for example, male blastocysts differ from females in terms of gene expression, mitochondrial DNA content, telomere length, DNA methylation, histone methylation and secretion of the signaling molecule, interferon-τ (Siqueira and Hansen 2016; Bermejo-Alvarez et al. 2010; Bermejo-Alvarez et al. 2008; Larson et al. 2001; Carvalheira et al. 2019). Female embryos have been reported to develop slower, undergo more apoptosis and have lower cell number than male embryos (Ghys et al. 2015; Oliveira et al. 2016). Transcriptional response to serum also depends on sex (Heras et al. 2016). Furthermore, actions of CSF2 differ between male and female embryos, with effects on development to the blastocyst stage reported to be greater for female embryos than male embryos (Siqueira and Hansen 2016) and effects on trophoblast elongation being positive for males and negative for females (Dobbs et al. 2014).

Resistance of the bovine embryo to stress has also been reported to be dependent upon sex. Female bovine embryos are more susceptible to deleterious effects of high concentrations of glucose in culture than are male embryos (Kimura et al. 2005). Sex differences in effects of oxidative stress depend on the culture medium, with male embryos being more resistant than female embryos in the presence of serum and less resistant in the absence (Dallemagne et al. 2018).

Here we evaluated whether CSF2 would increase resistance of bovine embryos to another stressful condition—cryopreservation. Bovine embryos are routinely stored in a cryopreserved state until transfer to a recipient female. Cryopreservation is achieved either by slow freezing using a programmable machine that controls the rate of cooling or by vitrification achieved by rapid cooling in the presence of high concentrations of cryoprotectants. Both procedures can result in a loss of embryo viability after thawing and reduced pregnancy success after transfer of embryos to recipients (Ferré et al. 2020). Damage to the embryo is multifactorial and includes ultrastructural damage from ice crystals (in slow freezing), cytotoxic effects of cryoprotectants, damage to lipid membranes caused by phase transition, lipid peroxidation, and induction of apoptosis after thawing (Ferré et al. 2020). It was hypothesized that cryosurvival of embryos treated with CSF2 from day 5 to 7 of development, a time when the embryo is progressing from the morula to blastocyst stage (Hansen and Tríbulo 2019) and when CSF2 can affect development, gene expression, and sensitivity to apoptosis (Moraes and Hansen 1997; Loureiro et al. 2009; Dobbs et al. 2013; Siqueira and Hansen 2016; Loureiro et al. 2011; Zolini et al. 2020), would increase the proportion of embryos that survived vitrification. The concentration of CSF2 used, 10 ng/mL, used was shown to protect embryos from heat shock (Loureiro et al. 2011) and induce expression of genes related to cell stress (Zolini et al. 2020). It was also hypothesized that male embryos would be more resistant to cryopreservation than female embryos. The endpoints used to determine survival were re-formation of the blastocoele of the blastocyst, which collapses during the cryopreservation process, and hatching of the blastocyst from the zona pellucida. These endpoints are commonly used to assess survival after cryopreservation (Vajta et al. 1998; Sudano et al. 2011; Lonergan et al. 2003; Block et al. 2010). Demonstrating a beneficial effect of CSF2 on cryosurvival of bovine blastocysts would strengthen the idea that CSF2 is a cytoprotective molecule for the preimplantation embryo and could mean that addition of CSF2 to culture medium could be one approach to improve cryosurvival of bovine embryos used commercially.

## Results

### Actions of CSF2 on competence of embryos to develop to the blastocyst stage

Treatment of embryos with CSF2 from day 5 to 7 of development caused a slight but significant increase in the percent of presumptive zygotes becoming blastocysts at day 7 of development (Table 1). There was no effect of CSF2 on the percent of blastocysts that were classified as advanced (i.e., either expanded, hatching or hatched) or that were undergoing hatching (i.e., classified as hatching or hatched) (Table 1).

A subset of blastocysts was sexed by PCR. The percent of blastocysts that were female was 39.9% (83/208) for vehicle and 42.7% (102/239) for CSF2. This difference was not significant (P = 0.578).

### Interactions between CSF2 and sex on survival of vitrified blastocysts after thawing and culture for 24 h (Experiment 1)

There were three objectives – to test whether CSF2 would increase survival of blastocysts to vitrification, as assessed by re-expansion of the blastocoele and initiation of hatching from the zona pellucida after 24 h of culture post-thawing, to determine whether male blastocysts survived vitrification better than female blastocysts, and to determine whether the effect of CSF2 depended on sex. Results are presented in Table 2. There was no effect of either CSF2, sex, or the interaction on the percent of blastocysts that re-expanded after thawing. The same was true for the percent of embryos that were hatching or hatched. Overall, 58.8% (108/262) of male blastocysts were hatching or hatched vs 50.8% (94/185) of female blastocysts.

### Effect of CSF2 on survival of vitrified blastocysts after thawing and prolonged culture for 72 h (Experiment 2)

The purpose of this experiment was to examine effects on longer-term survival of warmed blastocysts. The experiment was performed without sexing embryos because there was a lack of a CSF2 x sex interaction in the first experiment and because embryos that were degenerated after 24 h were difficult to harvest for sexing.

**Fig. 1:**
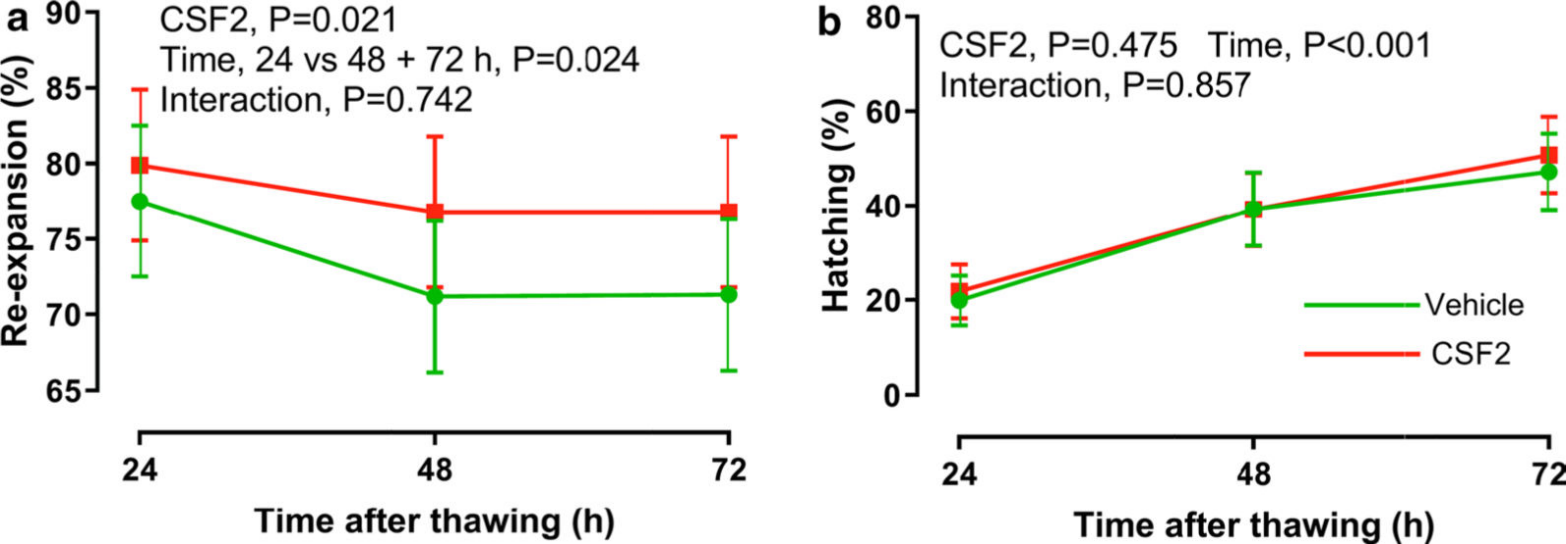
Effect of colony stimulating factor 2 (CSF2) on survival of vitrified blastocysts after warming and prolonged culture for 72 h. The total number of vitrified blastocysts that were examined after warming for re-expansion was n = 248 for vehicle and n = 242 for CSF2. The total number that were examined for hatching was n = 222 for vehicle and n = 217 for CSF2. Green lines and circles represent vehicle and red lines and squares represent CSF2. Data are least-squares means ± SEM. Levels of significance are indicated by text in each graph

Results for re-expansion are presented in Fig. 1a. The percent of blastocysts that re-expanded was higher for CSF2 than vehicle (main effect of CSF2 vs vehicle, P = 0.021). Overall, the percent of warmed blastocysts that was re-expanded was 73.3 ± 4.7% for vehicle and 77.8 ± 4.7% for CSF2 (averaged across times). The percent of blastocysts that re-expanded declined between 24 and 48 h and then remained constant (24 vs 48 + 72 h, P = 0.024). Although the interaction was not significant, the difference in re-expansion between CSF2 and vehicle was greater at 48 h (+ 5.6%) and 72 h (+ 5.5%) than at 24 h (+ 2.4%). Indeed, the difference between treatments at 24 h in Experiment 2 was similar to the difference at 24 h in Experiment 1 (+ 3.0%). For blastocysts treated with vehicle, percent re-expansion declined from 77.5% at 24 h to 71.2% and 71.3% at 48 and 72 h, respectively (a decline of 6.2% from 24 to 72 h). For CSF2-treated blastocysts, percent re-expansion declined from 79.9% at 24 h to 76.8% and 76.8% at 48 and 72 h, respectively (a decline of 3.1% from 24 to 72 h). The proportion of re-expanded blastocysts at 24 h that experienced blastocoel collapse by 72 h was lower (P = 0.053) for CSF2 (3.6%; 7/195) than for vehicle (8.2%; 16/195).

Results for hatching are presented in Fig. 1b. The percent of warmed blastocysts that were hatching or hatched increased with time (P < 0.001) but there was no effect of CSF2 (P = 0.475) or the interaction of CSF2 and time (P = 0.857) on hatching.

## Discussion

The present results indicate that CSF2 can promote survival of a fraction of blastocysts that were cryopreserved by vitrification. Although CSF2 did not cause a significant effect on the percent of blastocysts that re-established the blastocoele after 24 h of culture, CSF2 treatment before vitrification reduced the collapse of the blastocoele that occurred for a portion of the embryos that had experienced re-expansion at 24 h. The decline in the percent of embryos with a re-expanded blastocoele from 24 to 48 h was of a smaller magnitude for CSF2-treated blastocysts than for control blastocysts. Collapse of the blastocoele after prolonged culture is often observed for cryopreserved bovine blastocysts (Rizos et al. 2002; Mucci et al. 2006; Block et al. 2010) and reflects death of a proportion of blastocysts that initially survive after thawing.

The mechanism by which CSF2 improves blastocyst survival to cryopreservation was not studied but it is likely that CSF2 promotes activation of one or more pathways that stabilize cellular function during stress. Among the gene ontologies represented in genes whose expression in blastocysts was regulated by CSF2 were cell compromise and cell death and survival (Zolini et al. 2020). Moreover, CSF2 can block apoptosis in bovine blastocysts exposed to heat shock (Loureiro et al. 2011). Activation of apoptosis after thawing, as measured by group II caspase activity, was related to frequency of hatching in bovine blastocysts (Jousan et al. 2008). Moreover, blocking caspase activity with a chemical inhibitor increased percent re-expansion and hatching in warmed blastocysts after vitrification (Pero et al. 2018). It is possible that increased blastocyst cell number caused by CSF2, as reported in the mouse (Robertson et al. 2001) and human (Sjöblom et al. 1999), could increase survival. However, results are inconsistent as to whether CSF2 increases cell number in bovine blastocysts (Loureiro et al. 2009; Siqueira and Hansen 2016).

There was no effect of CSF2 on the percent of blastocysts that hatched or were undergoing hatching after vitrification. Therefore, the major effect of CSF2 was to prevent blastocyst death (as determined by loss of the blastocoele) rather than to enhance post-thawing development, as assessed by hatching, of those embryos that remained alive.

There was also no interaction between CSF2 and sex in embryo survival. Perhaps, sex does not affect cytoprotective effects of CSF2, unlike effects on development to the blastocyst stage (Siqueira and Hansen 2016) and effects on trophoblast elongation (Dobbs et al. 2014). One limitation of the study was that it was difficult to determine sex of degenerated blastocysts and this precluded analysis of blastocyst sex at 48 and 72 h. Given that the main effect of CSF2 occurred after 24 h, it is possible that interactions between CSF2 and sex would have been observed at 48 and 72 h.

As expected, CSF2 increased the percent of embryos becoming blastocysts in culture. Such an effect has been reported previously in the bovine (Moraes and Hansen 1997; Loureiro et al. 2009; Dobbs et al. 2013; Siqueira and Hansen 2016), human (Sjöblom et al. 1999), and pig (Kwak et al. 2012). The mechanism is not known although protection from cell stress is a conceivable one. In the cow, at least, the pro-developmental effects of CSF2 have been reported to occur in females and not males (Siqueira and Hansen 2016). The small magnitude of the effect of CSF2 on percent of embryos becoming blastocysts is also consistent with other experiments (Moraes and Hansen 1997; Loureiro et al. 2009; Dobbs et al. 2013; Siqueira and Hansen 2016). Indeed, the magnitude of effects of CSF2 have been reported to depend on the overall performance of the embryo culture system, with CSF2 increasing development when the development to the blastocyst rate in the control group is low, to have no effect when development in controls was moderate, and to be inhibitory when development was high (Dobbs et al. 2013). Such a result is also consistent with the pro-developmental effects of CSF2 being related to cell stress.

The concentration of CSF2 tested was 10 ng/mL. This concentration has been shown effective at increasing competence of the embryo to reach the blastocyst stage in vitro (Moraes and Hansen 1997; Loureiro et al. 2009; Dobbs et al. 2013; Siqueira and Hansen 2016), modify trophoblast elongation (Dobbs et al. 2014), increase embryo pregnancy rate after transfer to female recipients (Dobbs et al. 2013; Denicol et al. 2014), reduce the induction of apoptosis in Day 6 bovine embryos caused by heat shock (Loureiro et al. 2011) and regulate blastocyst gene expression (Zolini et al. 2020). Other concentrations of CSF2 were not tested but it is very probable that this concentration is sufficient to modify properties of the bovine embryo.

An important question is whether cryoprotective actions of CSF2 are of sufficient magnitude to justify inclusion of the embryokine in commercial protocols for cryopreservation of bovine embryos. In the mouse, CSF2 treatment before cryopreservation did not enhance survival following freezing but culture of embryos with CSF2 after thawing increased re-expansion of blastocysts (Papayannis et al. 2007). It is very likely that the effects of CSF2 seen here, which were of small magnitude and without an effect of CSF2 on hatching rate, were not large enough to have an impact of embryo survival after transfer. Moreover, treatments that improve indices of embryo survival to cryopreservation in vitro do not always result in increased pregnancy rates when embryos are transferred to recipients after thawing. An example is treatment with L-carnitine during culture (Zolini et al. 2020). Experiments are warranted to test whether CSF2 increases pregnancy rate of vitrified embryos after transfer to recipients and, if so, whether the improvement in pregnancy rate is greater than the positive effect of CSF2 when fresh embryos are transferred to females.

## Conclusion

Treatment with CSF2 from day 5 to 7 of development did not cause a significant effect on the percent of blastocysts that re-established the blastocoele after 24 h of culture but CSF2 reduced the collapse of the blastocoele that occurred for a portion of the embryos that had experienced re-expansion at 24 h. Thus, CSF2 can provide protection to a proportion of blastocysts from cryodamage caused by vitrification. This result is additional evidence of the ability of CSF2 to protect embryos from stress.

## Materials and methods

Approval from the Institutional Animal Care and Use Committee was not sought because animals were not used. Unless otherwise stated, all reagents were from ThermoFisher (Carlsbad, CA, USA) or Sigma-Aldrich (St. Louis, MO, USA).

### Embryo production

Ovaries were recovered from freshly-slaughtered cows from a local slaughterhouse. Ovaries were transported at 23 °C in 0.9% (w/v) NaCl supplemented with 10,000 units/mL penicillin and 10,000 μg/mL streptomycin. Once in the laboratory, ovaries with no cystic structures or follicles > 10 mm in diameter were selected for oocyte collection and rinsed several times to remove excess blood. Subsequent steps were all performed with solutions and media that were prewarmed to 38.5 °C.

All visible follicles that were 4–8 mm in diameter were bisected using a scalpel and then cumulus-oocyte complexes (COC) were released by swirling each ovary in a beaker containing oocyte collection medium (OCM) (Minitube, Verona, WI, USA). The fluid containing COC was filtered through a 100-μm Falcon cell strainer (ThermoFisher). The COC retained in the strainer were rinsed into a 100 mm × 100 mm square petri dish with gridlines using 20 mL of OCM and a 10-mL syringe connected to an 18-ga needle. Content of the dish was searched using a dissecting microscope and COC were removed using a wiretrol pipette (Drummond, Broomall, PA, USA).

Those COC with evenly granulated cytoplasm and three or more layers of cumulus cells were selected for in vitro maturation. Groups of 10 COC were matured in an incubator in a humidified atmosphere of 5% CO_2_ in air at 38.5 °C in 50-μl drops of a commercial maturation medium (IVF-Bioscience, Falmouth, Cornwall, UK) covered with mineral oil for 20–22 h.

Fertilization was achieved using frozen semen. Semen was warmed at 37 °C for 30 s. For each replicate, straws from three separate bulls were warmed and pooled together for fertilization. Different pools of bulls were used for each replicate. Sperm were purified from frozen-thawed semen using the PureSperm^®^ system gradient (Nidacon, Mölndal Sweden). The semen was gently placed over two layers (2 mL each) of 80% (bottom) and 40% (top) PureSperm 40/80 gradient in a 15-mL conical tube. Semen was first centrifuged at 1000 × g for 10 min to separate live sperm from dead sperm and to remove the diluent and seminal plasma. Then the resultant pellet at the bottom of the tube was placed in 10 mL of warmed HEPES-TALP (Tríbulo et al. 2019) in a 15-mL conical tube and a second centrifugation at 200 × g for 5 min was performed to wash the sperm. Sperm were resuspended in IVF-TALP medium (Tríbulo et al. 2019) previously equilibrated in an incubator in a humidified atmosphere of 5% CO_2_ in air at 38.5 °C and the sperm concentration adjusted to 16 × 10^6^ sperm/mL using a hemocytometer so that final concentration in the fertilization well was 1 × 10^6^/mL.

For fertilization, matured COC were rinsed 3 times with warmed HEPES-TALP and up to 300 matured COC were transferred to a fertilization dish containing 1.7 mL of IVF-TALP, 120 μl of sperm and 80 μl of PHE solution (0.5 mM penicillamine, 0.25 mM hypotaurine, 0.25 μM epinephrine), prepared as described elsewhere (Tríbulo et al. 2019). Gametes were co-incubated in a humidified atmosphere of 5% CO_2_ in air at 38.5° for 20–22 h.

After fertilization, cumulus cells were removed by placing presumptive zygotes (i.e., COC that had been placed with sperm in fertilization dishes) in a 2 mL tube containing 100 μl of 1000 U/mL hyaluronidase in 0.9% (w/v) NaCl and 500 μL HEPES-TALP. Cumulus cells were removed from the putative zygotes by agitation for 5 min using a vortex mixer. Putative zygotes were transferred using a wiretrol pipette in groups of 30 into 45-μl drops of Synthetic Oviduct Fluid-Bovine Embryo 2 (SOF-BE2) (Tríbulo et al. 2019) covered with mineral. Embryos were cultured for 7 days in a humidified atmosphere of 5% CO_2_, 5% O_2_, 90% N_2_ at 38.5 °C in an EVE model benchtop incubator (WTA, Cravinhos, SP, Brazil).

### Treatment of embryos with CSF2

Recombinant bovine CSF2 (Novartis, Basle, Switzerland) was stored at − 20 °C in aliquots of 1000 ng/mL in Dulbecco’s phosphate buffered saline containing 1 mg/mL bovine serum albumin (DPBS-BSA). The stock of CSF2 was thawed on the day of use and diluted 1:10 with SOF-BE2. The unused portion was discarded. Drops of cultured embryos were randomly assigned to receive either 10 ng/mL CSF2 or vehicle [a solution of 90% SOF-BE2 and 10% DPBS-BSA (v/v)]. Treatment consisted of adding either 5 μl of 100 ng/mL CSF2 or vehicle at day 5 after initiation of fertilization. Embryos were produced with or without CSF2 in a total of 25 replicates, where each replicate represents a group of about 200–400 COC collected on a single occasion. The total number of putative zygotes placed into culture was 2987 for vehicle and 3045 for CSF2.

### Vitrification

Blastocysts were harvested for vitrification at day 7 of development regardless of stage or quality grade. Procedures for vitrification followed those of Vajta et al. (1998). Incubation steps for vitrification were carried out in Nunclon Delta Treated 4-Well IVF dishes (ThermoFisher). All blastocysts from a specific treatment were placed in 800 μL holding medium (HM) in the first well. The HM consisted of a solution of TCM-199 with Hanks salts (Caisson Labs, Rexburg, Idaho, USA) and 10% (v/v) fetal bovine serum (FBS) (R&D Systems, Minneapolis, MN, USA). Embryos were then transferred to the second well containing 800 μl HM and held until further processing. In particular, groups of 4–5 blastocysts were placed in the third well containing 1000 μL equilibrium solution (ES) that consisted of HM (850 μL), ethylene glycol (75 μL) and dimethyl sulfoxide (75 μL). After 3 min, blastocysts were harvested in the smallest volume and placed in a single 15–20 μL drop of vitrification solution (VS) that was placed on the surface of the Nunclon dish. The VS consisted of HM containing 1 M sucrose, 16.5% (v/v) ethylene glycol + 16.5% (v/v) DMSO. Blastocysts were aspirated and expelled several times using a wiretrol pipette to ensure complete mixing to the VS. The wiretrol pipette was used to load the 4 to 5 blastocysts in 1 to 2 μL VS. The VS containing blastocysts was then expelled onto the surface of the vitrification dish to create a 1–2 μL drop. Once the 1–2 μl drop was made, an open pulled straw (OPS) (Minitube) was held at a 60–70° angle to the horizontal and the embryos were loaded into the straw through capillary action by touching the 1–2 μL drop with the narrow end of the OPS straw. After loading, the straw was immediately immersed in liquid nitrogen for vitrification. The entire time blastocysts remained in VS before vitrification was kept to a maximum of 25 s. Blastocysts were stored in liquid nitrogen until thawing for experiments.

### Warming and subsequent culture of vitrified blastocysts

Blastocysts were warmed using the procedure of Vajta et al. (1998). After removal from liquid nitrogen, the narrow tip of the OPS containing blastocysts was immersed in 1200 μL of a mixture of 67% (v/v) HM and 33% (v/v) sucrose medium (SM), which consisted of HM containing 1 M sucrose. Blastocysts were expelled by applying pressure to the other end of the straw with a fingertip. After expulsion, blastocysts were immediately transferred to a second well of 67% (v/v) HM + 33% (v/v) SM for 5 min. Blastocysts were then moved to a third well of 80% (v/v) HM + 20% (v/v) SM for 5 min and then to a well of 100% HM for 5 min.

After warming blastocysts and washing them through various mixtures of HM and SM as described above, groups of up to 15 blastocysts were cultured in 25 μL-drops of SOF-BE2 medium supplemented with 10% (v/v) FBS and 50 μM dithiothreitol and covered with mineral oil. Blastocysts were cultured in an EVE benchtop incubator (WTA) at 38.5 °C in a humidified atmosphere of 5% CO_2_, 5% O_2_, and 90% N_2_ for up to 72 h.

### Experiment 1: Interactions between CSF2 and sex on survival of vitrified blastocysts after thawing and culture for 24 h

Vitrified blastocysts produced in culture medium containing vehicle or CSF2 were cultured for 24 h as described above. At the end of culture, each blastocyst was classified based on whether the blastocoelic cavity reformed after thawing and whether the blastocyst was undergoing hatching (whether hatching or hatched from the zona pellucida). Subsequently, the blastocyst was sexed using polymerase chain reaction (PCR) as described below. The experiment was performed with blastocysts produced in 12 replicates. The original number of blastocysts that were vitrified and warmed was n = 268 for vehicle and n = 289 for CSF2, respectively. However, some embryos fell apart when being harvested for sexing and the final number of blastocysts for which data were available was n = 236 for vehicle and n = 264 for CSF2. The total number examined for hatching (after excluding blastocysts that were already hatching or hatched before vitrification) was n = 208 for vehicle and n = 239 for CSF2),

### Experiment 2: Effect of CSF2 on survival of vitrified blastocysts after thawing and prolonged culture for 72 h

The goal for Experiment 2 was to evaluate survival of warmed blastocysts over a longer time period than for Experiment 1. Blastocysts were produced in a total of 11 replicates. Embryos were cultured for 72 h as described above. Each blastocyst was examined at 24, 48 and 72 h and scored as to whether re-establishment of the blastocoele had occurred (yes/no) and whether the embryo had either hatched or was hatching (yes/no). Once an embryo had hatched, it is always classified as being hatched. However, an embryo that had re-established the blastocoele could subsequently be classified as not re-established if the blastocoele subsequently collapsed. The total number of vitrified blastocysts that were examined after thawing for re-expansion was n = 248 for vehicle and n = 242 for CSF2. The total number that were examined for hatching was n = 222 for vehicle and n = 217 for CSF2.

### Sexing of blastocysts

Procedures for DNA sexing of blastocysts were based on a previously-published technique using PCR with Y-specific and autosomal targets (Park et al. 2001). Primers were slightly different than those described earlier (Park et al. 2001) because of changes in published sequences for the genomic regions. Sequences of the BOV97M Y-specific primers were (forward) 5′-GATCACTATACATACACCACT-3′ and (reverse) 5′-AAGGCTATGCTACACAAATTCTG-3′. Sequences for primers that targeted an autosomal sequence on chromosome 19 were (forward) 5′TGGAAGCAAAGAACCCCGCT-3′ and (reverse) 5′TCGTGAGAAACCGCACCCTG-3′. The length of the male-specific amplification product is 143 base pairs and that of the targeted autosome amplification product is 217 base pairs.

Each blastocyst was harvested after 24 of culture and washed three times with diethyl pyrocarbonate-treated Dulbecco’s phosphate buffered saline containing 0.2% (w/v) polyvinylpyrrolidone (DPBS-PVP). Expanded and hatching blastocysts were incubated individually in drops of 50 μL Acid Tyrode’s solution (Sigma-Aldrich) for 3–4 min to remove the zona pellucida. Hatched blastocysts were incubated in 50 μL dissociation reagent (TrypLE^™^ Select, 10×, ThermoFisher) for 10 min to facilitate digestion during embryo lysis. This step was required for hatched blastocysts to ensure complete lysis. Blastocysts were then washed as mentioned above and then placed in 1 μl of resuspension buffer provided in the CellsDirect extraction kit (ThermoFisher), individually snap-frozen and stored at − 80 °C.

Each blastocyst was lysed by bringing to room temperature and adding 0.5 μL lysis enhancer from the CellsDirect extraction kit (ThermoFisher). The lysis enhancer was added to the tube containing the blastocyst while examining the embryo under a stereoscope to ensure the blastocyst was mixed with the lysis enhancer. Digestion proceeded at 70 °C for 20 min in a thermocycler. The tubes were mixed by tapping at 10 min. Subsequently, 1 μl of 100 μg/mL RNase A in nuclease-free water (Qiagen, Germantown, MD, USA) was added and samples were incubated at 37 °C for 30 min.

For PCR, mastermix was prepared as follows: 4 μL of 5× Green GoTap Flexi Buffer (Promega, Madison, WI, USA), 4 μL of 25 mM MgCl_2_ (Promega), 1 μL of 10 mM dNTP (Invitrogen, ThermoFisher), 0.4 μL of 10 μM sex primers (forward + reverse), 0.2 μL of GoTaq hot start polymerase (Promega), and 6.6.μL nuclease-free water. A total of 16.2 μl of mastermix was added to each tube containing a single blastocyst. The final volume was ~ 19 μl. As a positive control, 16.2 μl of mastermix was added to a tube containing 2.8 μl of 10 ng/μL genomic DNA isolated from blood of male and female cattle. The negative control consisted of 16.2 μl of mastermix added to a tube containing 2.8 μL nuclease-free water. The first round of PCR for sex primers was performed using the following protocol: 95 °C, 5 min; (95 °C, 15 s; 58 °C, 15 s; 72 °C, 15 s) X 20; 72 °C, 10 min. After addition of 1 μl of 4 μM of each autosomal primer to each tube, a second round of PCR was performed using the following protocol: 95 °C, 5 min; (95 °C, 15 s; 58 °C, 15 s; 72 °C, 15 s) X 17; 72 °C, 10 min.

Sexing was achieved by examining the size of amplicons as determined by electrophoresis of reaction products on 2% (w/v) agarose gels. The gels were prepared by adding 2 g low EEO agarose (ThermoFisher) to 100 mL of 2 M Tris, 1 M acetic acid and 0.05 M ethylenediamine tetraacetic acid (TAE) buffer 1X (diluted from 10X, Sigma-aldrich). The poured gel was submerged beneath the TAE buffer. PCR products were prepared for electrophoresis by adding 2 μL of Diamond Nucleic Acid Dye (Promega) with 8 μL of PCR product. Samples were loaded into the wells of the gel and electrophoresis was performed at 100 V and 160 mA for 40–60 min. Finally, images were captured using a photoimager and subsequently analyzed for presence of amplicons for autosomal primers (a positive control; present for all blastocysts) and the Y-specifc primers (present in males only).

### Statistical analysis

Data on re-expansion was analyzed for all warmed blastocysts. For hatching after thawing however, blastocysts that were hatching and hatched before vitrification were removed from the analysis.

Data on development, re-expansion and hatching were analyzed by logistic regression fitted to a binomial distribution using the GLIMMIX procedure of the Statistical Analysis System version 9.4 (SAS Institute, Cary, NC, USA). Models included various fixed effects of treatment, time (for Experiment 2) and random effect of replicate. The contrast statement was used to separate time effects into two orthogonal contrasts: 24 h vs 48 + 72 and 48 h vs 72 h. Effect of CSF2 on the percent of re-expanded blastocysts at 24 h that experienced collapse of the blastocoel at 72 h was determined by chi-square analysis.

## Supplementary Material

Additional File 1

## Figures and Tables

**Table 1:** Effect of 10 ng/mL colony stimulating factor 2 (CSF2) from day 5 to 7 on development of putative zygotes to the blastocyst stage

Treatment	No. of replicates	No. of presumptive zygotes	Zygotes that became blastocysts (%)^a^	Blastocysts that were advanced (%)^abc^	Blastocysts that were hatched or hatching (%)^ac^

Vehicle	25	2987	22.1 ± 1.4	57.2 ± 3.2	9.8 ± 1.4
CSF2	25	3045	24.7 ± 1.5	58.8 ± 3.1	12.2 ± 1.5
P			0.024	0.558	0.140

a Data are least-squares means ± SEM

b Advanced blastocysts were those that were expanded, hatching or hatched

c Calculated as a percent of all blastocysts

**Table 2:** Effect of colony stimulating factor 2 (CSF2) and sex on survival of blastocysts following vitrification as evaluated by re-expansion of the blastocoele and initiation of hatching from the zona pellucida after 24 h of culture post-thawing

Treatment	Sex	Number of blastocysts	Re-expansion (%)^a^	Number of blastocysts^b^	Hatching (%)^a^

Vehicle	Female	94	84.0 ± 3.8	83	52.5 ± 6.9
CSF2	Female	115	87.8 ± 3.0	102	51.1 ± 5.8
Vehicle	Male	142	82.4 ± 3.2	125	58.8 ± 6.5
CSF2	Male	149	84.6 ± 3.0	137	58.7 ± 5.8
P values					
CSF2			0.525		0.882
Sex			0.582		0.160
CSF2 x sex			0.810		0.902

a Data are least-squares means ± SEM

b Number is lower than for re-expansion because embryos that were hatching or hatched before vitrification were excluded

## References

[R1] Bermejo-Alvarez P, Rizos D, Rath D, Lonergan P, Gutierrez-Adan A. Epigenetic differences between male and female bovine blastocysts produced in vitro. Physiol Genomics. 2008;32:264–72. 10.1152/physiolgenomics.00234.2007. 17986520

[R2] Bermejo-Alvarez P, Rizos D, Rath D, Lonergan P, Gutierrez-Adan A. Sex determines the expression level of one third of the actively expressed genes in bovine blastocysts. Proc Natl Acad Sci USA. 2010;107:3394–9. 10.1073/pnas.0913843107. 20133684 PMC2840439

[R3] Block J, Bonilla L, Hansen PJ. Efficacy of in vitro embryo transfer in lactating dairy cows using fresh or vitrified embryos produced in a novel embryo culture medium. J Dairy Sci. 2010;93:5234–42. 10.3168/jds.2010-3443. 20965338

[R4] Carvalheira LR, Tríbulo P, Borges ÁM, Hansen PJ. Sex affects immunolabeling for histone 3 K27me3 in the trophectoderm of the bovine blastocyst but not labeling for histone 3 K18ac. PLoS ONE. 2019;14:e0223570. 10.1371/journal.pone.0223570. 31600298 PMC6786533

[R5] Chin PY, Macpherson AM, Thompson JG, Lane M, Robertson SA. Stress response genes are suppressed in mouse preimplantation embryos by granulocyte-macrophage colony-stimulating factor (GM-CSF). Hum Reprod. 2009;24:2997–3009. 10.1093/humrep/dep307. 19737804

[R6] Dallemagne M, Ghys E, De Schrevel C, Mwema A, De Troy D, Rasse C, Donnay I. Oxidative stress differentially impacts male and female bovine embryos depending on the culture medium and the stress condition. Theriogenology. 2018;117:49–56. 10.1016/j.theriogenology.2018.05.020. 29859336

[R7] Denicol AC, Block J, Kelley DE, Pohler KG, Dobbs KB, Mortensen CJ, Ortega MS, Hansen PJ. The WNT signaling antagonist Dickkopf-1 directs lineage commitment and promotes survival of the preimplantation embryo. FASEB J. 2014;28:3975–86. 10.1096/fj.14-253112. 24858280 PMC5395727

[R8] Dobbs KB, Khan FA, Sakatani M, Moss JI, Ozawa M, Ealy AD, Hansen PJ. Regulation of pluripotency of inner cell mass and growth and differentiation of trophectoderm of the bovine embryo by colony stimulating factor. Biol Reprod. 2013;89:141. 10.1095/biolreprod.113.113183. 24198123

[R9] Dobbs KB, Gagné D, Fournier E, Dufort I, Robert C, Block J, Sirard MA, Bonilla L, Ealy AD, Loureiro B, Hansen PJ. Sexual dimorphism in developmental programming of the bovine preimplantation embryo caused by colony-stimulating factor 2. Biol Reprod. 2014;91:80. 10.1095/biolreprod.114.121087. 25078682

[R10] Ferré LB, Kjelland ME, Taiyeb AM, Campos-Chillon F, Ross PJ. Recent progress in bovine in vitro-derived embryo cryotolerance: Impact of in vitro culture systems, advances in cryopreservation and future considerations. Reprod Domest Anim. 2020;1:1. 10.1111/rda.13667. 32144939

[R11] Ghys E, Dallemagne M, De Troy D, Sauvegarde C, Errachid A, Donnay I. Female bovine blastocysts are more prone to apoptosis than male ones. Theriogenology. 2015;85:591–600. 10.1016/j.theriogenology.2015.09.050. 26506912

[R12] Hansen PJ, Tríbulo P. Regulation of present and future development by maternal regulatory signals acting on the embryo during the morula to blastocyst transition - insights from the cow. Biol Reprod. 2019;101:526–37. 10.1093/biolre/ioz030. 31220231 PMC8127039

[R13] Heras S, De Coninck DI, Van Poucke M, Goossens K, Bogado Pascottini O, Van Nieuwerburgh F, Deforce D, De Sutter P, Leroy JL, Gutierrez-Adan A, Peelman L, Van Soom A. Suboptimal culture conditions induce more deviations in gene expression in male than female bovine blastocysts. BMC Genomics. 2016;17:72. 10.1186/s12864-016-2393-z. 26801242 PMC4724126

[R14] Jousan FD, Castro E, Paula LA, Brad AM, Roth Z, Hansen PJ. Relationship between group II caspase activity of bovine preimplantation embryos and capacity for hatching. J Reprod Dev. 2008;54:217–20. 18277054 10.1262/jrd.19175

[R15] Kannampuzha-Francis J, Denicol AC, Loureiro B, Kaniyamattam K, Ortega MS, Hansen PJ. Exposure to colony stimulating factor 2 during preimplantation development increases postnatal growth in cattle. Mol Reprod Dev. 2015;82:892–7. 10.1002/mrd.22533. 26227079

[R16] Kimura K, Spate LD, Green MP, Roberts RM. Effects of D-glucose concentration, D-fructose, and inhibitors of enzymes of the pentose phosphate pathway on the development and sex ratio of bovine blastocysts. Mol Reprod Dev. 2005;72:201–7. 15968626 10.1002/mrd.20342

[R17] Kwak SS, Jeung SH, Biswas D, Jeon YB, Hyun SH. Effects of porcine granulocyte-macrophage colony-stimulating factor on porcine in vitro-fertilized embryos. Theriogenology. 2012;77:1186–97. 10.1016/j.theriogenology.2011.10.025. 22153263

[R18] Larson MA, Kimura K, Kubisch HM, Roberts RM. Sexual dimorphism among bovine embryos in their ability to make the transition to expanded blastocyst and in the expression of the signaling molecule IFN-tau. Proc Natl Acad Sci USA. 2001;98:9677–82. 11481449 10.1073/pnas.171305398 PMC55511

[R19] Lonergan P, Rizos D, Kanka J, Nemcova L, Mbaye AM, Kingston M, Wade M, Duffy P, Boland MP. Temporal sensitivity of bovine embryos to culture environment after fertilization and the implications for blastocyst quality. Reproduction. 2003;126:337–46. 10.1530/reprod/126.3.337. 12968941

[R20] Loureiro B, Bonilla L, Block J, Fear JM, Bonilla AQ, Hansen PJ. Colony-stimulating factor 2 (CSF-2) improves development and posttransfer survival of bovine embryos produced in vitro. Endocrinology. 2009;150:5046–54. 10.1210/en.2009-0481. 19797121 PMC2775977

[R21] Loureiro B, Oliveira LJ, Favoreto MG, Hansen PJ. Colony-stimulating factor 2 inhibits induction of apoptosis in the bovine preimplantation embryo. Am J Reprod Immunol. 2011;65:578–88. 10.1111/j.1600-0897.2010.00953.x. 21223422

[R22] de Moraes AA, Hansen PJ. Granulocyte-macrophage colony-stimulating factor promotes development of in vitro produced bovine embryos. Biol Reprod. 1997;57:1060–5. 10.1095/biolreprod57.5.1060. 9369171

[R23] de Moraes AA, Paula-Lopes FF, Chegini N, Hansen PJ. Localization of granulocyte-macrophage colony-stimulating factor in the bovine reproductive tract. J Reprod Immunol. 1999;42:135–45. 10.1016/s0165-0378(98)00075-8. 10221736

[R24] Mucci N, Aller J, Kaiser GG, Hozbor F, Cabodevila J, Alberio RH. Effect of estrous cow serum during bovine embryo culture on blastocyst development and cryotolerance after slow freezing or vitrification. Theriogenology. 2006;65:1551–622. 16229883 10.1016/j.theriogenology.2005.08.020

[R25] Oliveira CS, Saraiva NZ, de Lima MR, Oliveira LZ, Serapião RV, Garcia JM, Borges CA, Camargo LS. Cell death is involved in sexual dimorphism during preimplantation development. Mech Dev. 2016;139:42–50. 10.1016/j.mod.2015.12.001. 26752320

[R26] Papayannis M, Eyheremendy V, Sanjurjo C, Blaquier J, Raffo FG. Effect of granulocyte-macrophage colony stimulating factor on growth, resistance to freezing and thawing and re-expansion of murine blastocysts. Reprod Biomed Online. 2007;14:96–101. 10.1016/s1472-6483(10)60770-5. 17207341

[R27] Park JH, Lee JH, Choi KM, Joung SY, Kim JY, Chung GM, Jin DI, Im KS. Rapid sexing of preimplantation bovine embryo using consecutive and multiplex polymerase chain reaction (PCR) with biopsied single blastomere. Theriogenology. 2001;55:1843–53. 10.1016/s0093-691x(01)00526-x. 11414489

[R28] Pero ME, Zullo G, Esposito L, Iannuzzi A, Lombardi P, De Canditiis C, Neglia G, Gasparrini B. Inhibition of apoptosis by caspase inhibitor Z-VAD-FMK improves cryotolerance of in vitro derived bovine embryos. Theriogenology. 2018;108:127–35. 10.1016/j.theriogenology.2017.11.031. 29207293

[R29] Rizos D, Ward F, Duffy P, Boland MP, Lonergan P. Consequences of bovine oocyte maturation, fertilization or early embryo development in vitro versus in vivo: implications for blastocyst yield and blastocyst quality. Mol Reprod Dev. 2002;61:234–48. 11803560 10.1002/mrd.1153

[R30] Robertson SA, Sjöblom C, Jasper MJ, Norman RJ, Seamark RF. Granulocyte-macrophage colony-stimulating factor promotes glucose transport and blastomere viability in murine preimplantation embryos. Biol Reprod. 2001;64:1206–15. 10.1095/biolreprod64.4.1206. 11259269

[R31] Siqueira LG, Hansen PJ. Sex differences in response of the bovine embryo to colony-stimulating factor 2. Reproduction. 2016;152:645–54. 10.1530/REP-16-0336. 27601717 PMC5097130

[R32] Sjöblom C, Wikland M, Robertson SA. Granulocyte–macrophage colony-stimulating factor promotes human blastocyst development in vitro. Hum Reprod. 1999;14:3069–76. 10.1093/humrep/14.12.3069. 10601098

[R33] Sudano MJ, Paschoal DM, Rascado TS, Magalhães LC, Crocomo LF, Lima-Neto JF, Landim-Alvarenga FC. Lipid content and apoptosis of in vitro-produced bovine embryos as determinants of susceptibility to vitrification. Theriogenology. 2011;75:1211–20. 10.1016/j.theriogenology.2010.11.033. 21247620

[R34] Tríbulo P, Siqueira LGB, Oliveira LJ, Scheffler T, Hansen PJ. Identification of potential embryokines in the bovine reproductive tract. J Dairy Sci. 2018;101:690–704. 10.3168/jds.2017-13221. 29128220

[R35] Tríbulo P, Rivera RM, Ortega Obando MS, Jannaman EA, Hansen PJ. Production and culture of the bovine embryo. Methods Mol Biol. 2019;2006:115–29. 10.1007/978-1-4939-9566-0_8. 31230276

[R36] Vajta G, Holm P, Kuwayama M, Booth PJ, Jacobsen H, Greve T, Callesen H. Open pulled straw (OPS) vitrification: a new way to reduce cryoinjuries of bovine ova and embryos. Mol Reprod Dev. 1998;51:53–8. 10.1002/(SICI)1098-2795(199809)51:1&lt;53:AID-MRD6&gt;3.0.CO;2-V. 9712317

[R37] Zolini AM, Carrascal-Triana E, RuizdeKing A, Hansen PJ, AlvesTorres CA, Block J. Effect of addition of l-carnitine to media for oocyte maturation and embryo culture on development and cryotolerance of bovine embryos produced in vitro. Theriogenology. 2019;133:135–43. 10.1016/j.theriogenology.2019.05.005. 31091484

[R38] Zolini AM, Block J, Rabaglino MB, Tríbulo P, Hoelker M, Rincon G, Bromfield JJ, Hansen PJ. Molecular fingerprint of female bovine embryos produced in vitro with high competence to establish and maintain pregnancy. Biol Reprod. 2020;102:292–305. 10.1093/biolre/ioz190. 31616926 PMC7331872

